# Epidemiological and Genetic Characteristics of Sapovirus in Shandong, China, 2022–2023

**DOI:** 10.3390/v17040469

**Published:** 2025-03-26

**Authors:** Mingxin Guo, Meijia Li, Ti Liu, Wenkui Sun, Kaige Du, Shuopeng Yang, Zhongyan Fu, Zengqiang Kou

**Affiliations:** 1 School of Public Health and Health Management, Shandong First Medical University, Shandong Academy of Medical Sciences, Jinan 250117, China; gmx15610398290@163.com (M.G.); 15666250190@163.com (M.L.); 2 Shandong Provincial Center for Disease Control and Prevention, Jinan 250014, China; liuti1204@126.com (T.L.); wenkuisun@163.com (W.S.); 18369607969@163.com (K.D.); yyysp123456@163.com (S.Y.); 3 Shandong Provincial Key Laboratory of Intelligent Monitoring, Early Warning, Prevention and Control for Infectious Diseases, Jinan 250014, China

**Keywords:** sapovirus, epidemiology, genotypes, genome analysis

## Abstract

Sapovirus (SaV) is a major pathogen responsible for acute gastroenteritis (AGE), and its incidence has been increasing in recent years. This study investigates the prevalence and the genetic characteristics of SaV in Shandong Province during 2022–2023, based on a surveillance network covering all age groups. Samples were obtained from a viral diarrhea surveillance network in Shandong Province during 2022–2023. SaVs were identified through quantitative reverse-transcription polymerase chain reaction (RT-qPCR). PCR amplification and Sanger sequencing were performed on positive samples, and whole-genome sequencing was conducted using metagenomic sequencing technology. Sequence analysis was conducted using BioEdit 7.0.9.0 and MEGA X, while statistical analysis was performed with SPSS 26.0. A total of 157 SaV-positive cases were identified, resulting in a positivity rate of 1.12%. The positivity rate for SaV was 0.75% in 2022 and it increased significantly to 1.42% in 2023. The highest positivity rates for both 2022 and 2023 were observed in November. The highest positivity rate was observed in the 3–5-year-old age group. In 2022, Dongying City had the highest positivity rate, while Zaozhuang City exhibited the highest rate in 2023. The incidence of vomiting in SaV-positive patients was significantly higher compared to SaV-negative patients (*P* = 0.002). Eight genotypes were identified in both the VP1 and RdRp regions. The complete genome sequence analysis of a GI.3 strain showed that NS1 (5.88%, 4/68) was the region most prone to amino acid variation, followed by VP2 (5.45%, 9/165) within the same genotype. SaV infections are more prevalent in cold weather, with young children being particularly susceptible. The SaV positivity rate in 2023 increased significantly accompanied by an increased diversity of genotypes, compared to that of 2022. The NS1 region exhibits the biggest variation within the same genotype, indicating that more attention should be paid to other regions besides VP1 in the future study. Ongoing surveillance of SaV is recommended for effective prevention and control.

## 1. Introduction

Sapovirus (SaV) was first identified in human fecal samples in 1976 using electron microscopy [1]. It is a significant pathogen responsible for acute gastroenteritis (AGE), characterized by clinical symptoms such as fever, nausea, vomiting, diarrhea, abdominal pain, and abdominal cramps [2]. SaV can infect individuals across all age groups, with children being the most commonly affected population [3,4,5]. SaV can cause both sporadic infections and outbreaks [6,7]. In recent years, following the end of the COVID-19 pandemic, an increase in SaV incidence was observed [8]. Furthermore, studies from countries that have incorporated the rotavirus vaccine into their immunization programs showed an increasing trend of SaV infection [9].

A member of the Caliciviridae family, SaV has a single-stranded, positive-sense RNA genome approximately 7.1–7.7 kb in length, containing 2–3 open reading frames (ORFs). Recent studies have established a dual-typing classification system for SaV based on the complete nucleotide sequences of the RdRp and VP1 regions [10]. This system identifies 34 genogroups and 24 P-genogroups. Among the genogroups that infect humans (GI, GII, GIV, and GV), 20 genotypes (GI.1-7, GII.1-8, GII.NA1, GIV.1, GV.1-2, and GV.NA1) and 21 P-genotypes (GI.P1-P3, GI.P5-P7, GII.P1-P10, GII.PNA1-PNA2, GV.P1-P2, and GV.PNA1) have been identified.

In Shandong Province, a viral diarrhea surveillance network covering all age groups was founded in 2016 and SaV was included in the surveillance in 2022. Each county investigated 3–5 cases of viral diarrhea per month and collected fecal samples to obtain pathological data. This study analyzed infections and the genetic characteristics of SaV in Shandong Province during 2022–2023. The epidemiological and pathogenic characteristics of SaV were identified.

## 2. Materials and Methods

### 2.1. Sample Source

A viral diarrhea surveillance network was implemented in 136 counties spanning all 16 cities of Shandong Province. Each county was monitored for three to five cases of viral diarrhea per month. Demographic data were collected through epidemiological surveys, and key clinical symptoms—including fever, nausea, vomiting, diarrhea, and abdominal pain—were systematically documented. Furthermore, fecal samples were systematically collected. Over the surveillance period from 1 January 2022 to 31 December 2023, a total of 14,011 case records and corresponding stool specimens were collected, of which 157 cases tested positive for SaV infection.

### 2.2. RNA Extraction and SaV RT-qPCR Detection

Fecal samples were processed to a 10% stool suspension using a Phosphate-Buffered Saline (PBS) buffer. Nucleic acid extraction was performed by 136 county-level Centers for Disease Control and Prevention (CDCs)in Shandong Province using several different nucleic acid extraction kits, followed by SaV detection. The extracted nucleic acids were subsequently transferred to municipal CDCs for SaV detection to ensure the accuracy of the results. SaV detection was performed using a commercial RT-qPCR kit (MABSKY, Shenzhen, China). The nucleic acid and test results were ultimately sent to the provincial CDC for compilation and analysis.

### 2.3. Genotype Identification

The RdRp and VP1 regions of SaV-positive samples were subsequently amplified according to Oka et al. [3]. The RdRp region of SaV-positive samples was amplified by RT-PCR using the SuperScript™ III One-Step RT-PCR with Platinum™ Taq kit (Invitrogen, Thermo Fisher Scientific, Waltham, MA, USA). The PCR amplification mixture contained 5 μL of template RNA, 15 μL of 2× reaction mix, 0.9 μL of each forward primer p290h (5′-GATTACTCCAGGTGGGACTCCAC-3′), p290i (5′-GATTACTCCAGGTGGGACTCAAC-3′) and p290j (5′-GATTACTCCAGGTGGGATTCAAC-3′), p290k (5′-GATTACTCCAGGTGGGATTCCAC-3′), 1.2 μL of each reverse primer p289h (5′-TGACGATTTCATCATCACCATA-3′) and p289i (5′-TGACGATTTCATCATCCCCGTA-3′), 1.2 μL of Taq mix, and 2.8 μL of RNase-free water. The amplification conditions were 50 °C for 30 min reverse transcription, initial denaturation at 94 °C for 2 min, then 38 cycles of 94 °C for 30 s denaturation, 55 °C for 30 s for primer annealing, 68 °C for 1 min for extension, and finally 68 °C for 7 min for late extension. The expected PCR product size was 331 bp. The VP1 region of SaV-positive samples was amplified through an RT-nested PCR protocol. The first round of amplification was performed using the same kit as for the RdRp region. The PCR reaction mixture contained 5 μL of template RNA, 10 μL of 2× reaction mix, 0.6 μL of each forward primer SaV124F (5′-GAYCASGCTCTCGCYACCTAC-3′) and SaV1F (5′-TTGGCCCTCGCCACCTAC-3′), SaV5F (5′-TTTGAACAAGCTGTGGCATGCTAC-3′) and each reverse primer SV-R13 (5′-GGTGANAYNCCATTKTCCAT-3′) and SV-R14 (5′-GGTGAGMMYCCATTCTCCAT-3′), 0.8 μL of Taq mix, and 1.2 μL of RNase-free water. The thermal cycling conditions were as follows: initial denaturation at 94 °C for 3 min, followed by 35 cycles of denaturation at 94 °C for 30 s, annealing at 50 °C for 30 s, and extension at 72 °C for 1 min, with a final extension at 72 °C for 7 min. The second round of amplification was performed using the Platinum™ Taq DNA Polymerase kit (Invitrogen). The PCR reaction mixture contained 2 μL of 10× PCR Buffer-Mg, 0.6 μL of 50Mm MgCl_2_, 0.4 μL of 10Mm dNTP mix, 0.4 μL of forward primer 1245Rfwd (5′-TAGTGTTTGARATGGAGGG-3′), reverse primer SV-R2 (5′- GWGGGRTCAACMCCWGGTGG-3′), 0.08 μL of Taq DNA Polymerase, 1 μL of the first-round PCR product, and 15.12 μL of RNase-free water. The thermal cycling conditions were as follows: reverse transcription at 50 °C for 30 min, initial denaturation at 94 °C for 2 min, followed by 40 cycles of denaturation at 94 °C for 30 s, annealing at 55 °C for 30 s, and extension at 68 °C for 1 min, with a final extension at 68 °C for 7 min. The expected PCR product size was 440 bp. The PCR products were sent to Sangon Biotechnology (Shanghai) Co., Ltd. for Sanger sequencing. The nucleotide sequences were used for genotype identification through phylogenetic analysis with reference sequences by Zhao et al. [10].

### 2.4. Next-Generation Sequencing (NGS)

Complete-genome sequencing of SaV was conducted using metagenomic sequencing technology. Library preparation was carried out with the VAHTS Universal V8 RNA-seq Library Prep Kit for Illumina (Vazyme, Nanjing, China) following the manufacturer’s protocol. The prepared library was sequenced on an Illumina NextSeq 2000 platform (Illumina, San Diego, CA, USA) for high-throughput analysis. Raw sequencing data were assembled using the self-built workflow in CLC Genomics Workbench V21.0.2 (https://www.qiagenbioinformatics.com) (accessed on 18 October 2024), and the finalized sequences were deposited in the GenBank database (PV014972).

### 2.5. Sequence Analysis

The nucleotide and amino acid sequences were aligned using the ClustalW Multiple Alignment tool in BioEdit 7.0.9.0 [11]. Phylogenetic trees were constructed to illustrate genetic relationships using the Maximum Likelihood (ML) method with the Kimura 2-parameter nucleotide substitution model in MEGA X [12]. The reliability of the phylogenetic analysis was assessed with bootstrap values based on 1000 replicates.

### 2.6. Statistical Analysis

Epidemiological data were compiled using Excel 2021 (https://www.microsoft.com) (accessed on 1 January 2023), while statistical analyses were conducted with SPSS 26.0 (https://www.ibm.com/products/spss-statistics) (accessed on 1 January 2023). Categorical data were presented as rates or proportions. Group comparisons were performed using the chi-square test, with a significance level set at α = 0.05. *P* < 0.05 was considered statistically significant.

## 3. Results

This study analyzed 14,011 cases and identified 157 SaV-positive cases, resulting in a positivity rate of 1.12%.

### 3.1. Temporal, Age, and Spatial Distribution

**Figure 1:**
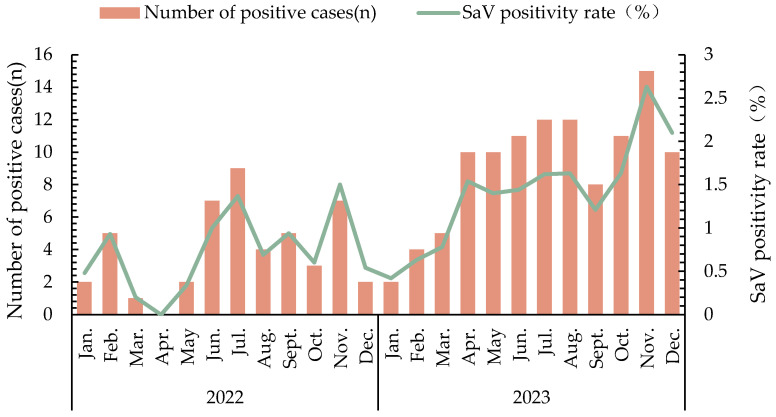
Temporal distribution of SaV infections in 2022–2023.

The positivity rate for SaV was 0.75% (47/6,290) in 2022 and it increased significantly to 1.42% (110/7,721) in 2023 (*χ^2^* = 14.358, *P* < 0.05). Except for April 2022, SaV was identified in all other months. The highest positivity rates occurred in November of both years, reaching 1.50% in 2022 and 2.63% in 2023, as detailed in Table 1 and Figure 1.

SaV was detected across all age groups. The highest positivity rate, 2.37%, was observed in the 3–5-year-old age group, primarily comprising preschool children, followed by 1.89% in the 12–17-year-old age group. The differences in SaV positivity rates among age groups were statistically significant (*χ^2^* = 47.289, *P* < 0.05), as detailed in Table 2.

**Figure 2:**
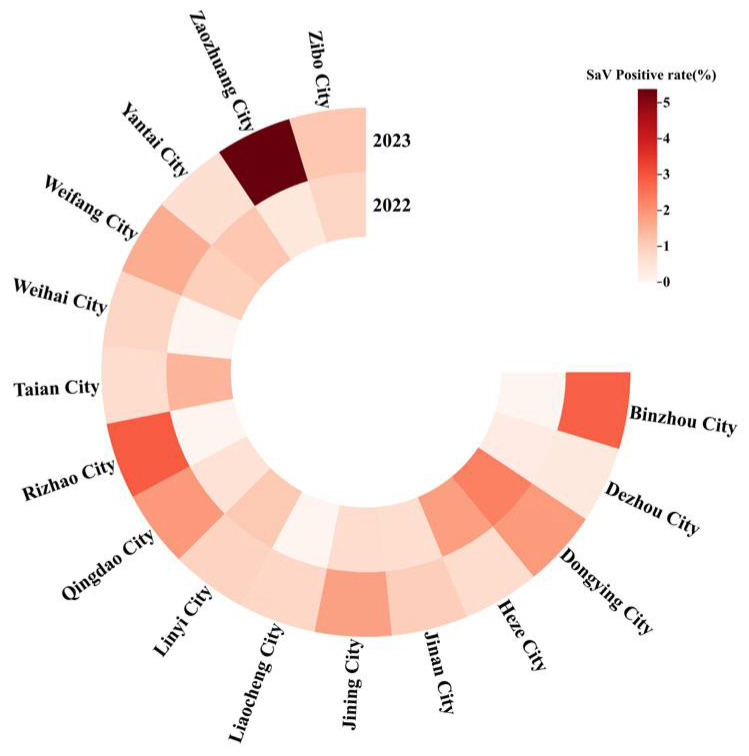
Detection of SaV across 16 cities in Shandong, 2022–2023. The color gradient in the legend, ranging from light to dark, represents an increasing SaV positivity rate, with lighter shades indicating lower rates and darker shades denoting higher rates.

The detection of SaV across cities in Shandong Province from 2022 to 2023 is shown in Figure 2. In 2022, Dongying City exhibited the highest positivity rate, at 2.30%. In 2023, Zaozhuang City recorded the highest positivity rate, reaching 5.38%.

### 3.2. Clinical Manifestation

Among the 14,011 cases, 195 records were excluded due to incompleteness or invalidity, leaving 13,816 cases for analysis. Of these, 11,458 (83%) were outpatient cases, and 2358 (17%) were inpatient cases. The SaV positivity rate was 1.09% for outpatients and 1.27% for inpatients. A comparison of SaV positivity rates between outpatient and inpatient cases revealed no statistically significant difference (*χ^2^* = 0.580, *P* = 0.446), as shown in Table 3.

As for symptoms, the incidence of vomiting is significantly higher in SaV-positive patients, compared to SaV-negative patients (*P* = 0.002). However, no statistically significant differences were observed in the incidence of other symptoms between the two groups (Table 4).

### 3.3. Genotype Distribution

**Figure 3:**
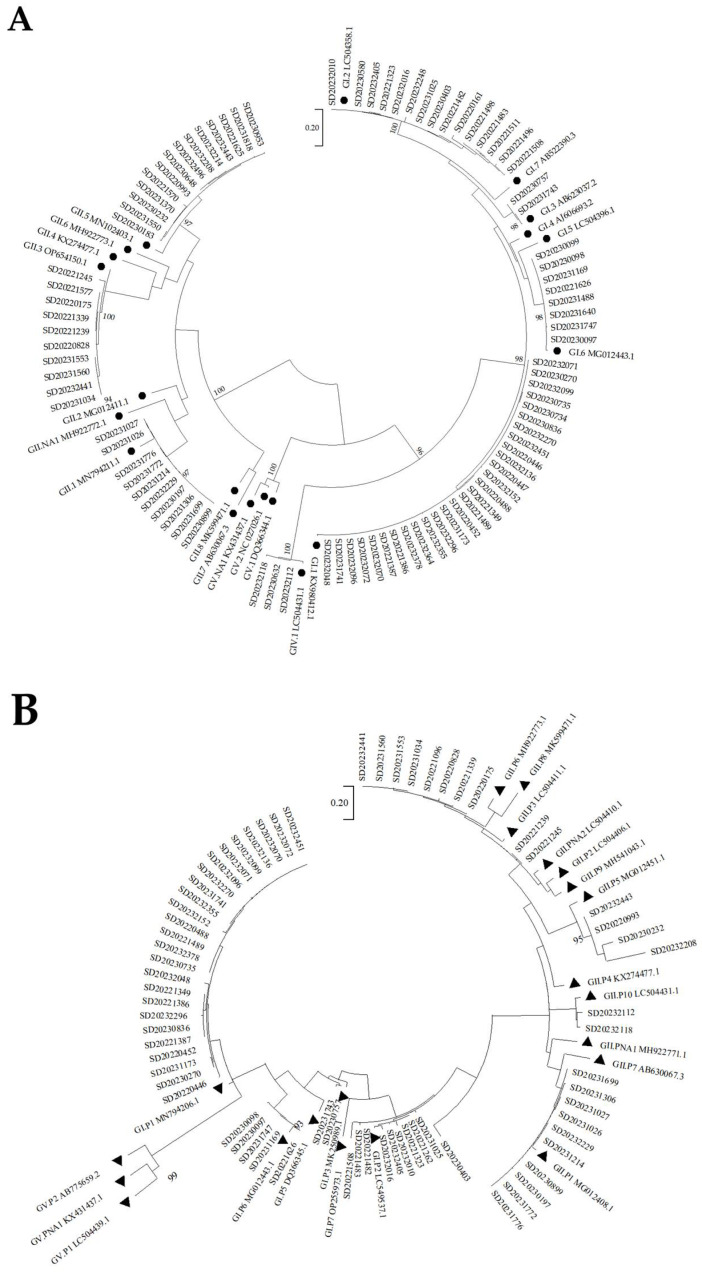
Phylogenetic analysis of nucleotide sequences in the SaV VP1 and RdRp regions. (**A**) ● indicates the reference sequences of VP1 region. The sequence length was 274 bp of the partial VP1 region. (**B**) ▲ indicates the reference sequences of RdRp region. The sequence length was 132 bp of the partial RdRp region.

Sequencing of the VP1 region was successfully performed on 90 samples, revealing eight genotypes: GI.1 (28 strains), GI.2 (15 strains), GI.3 (2 strains), GI.6 (8 strains), GII.1 (10 strains), GII.3 (10 strains), GII.5 (14 strains), and GIV.1 (3 strains). Sequencing of the RdRp region was successful for 68 samples, identifying eight P-genotypes: GI.P1 (25 strains), GI.P2 (10 strains), GI.P3 (2 strains), GI.P6 (5 strains), GII.P1 (10 strains), GII.P3 (10 strains), GII.P5 (4 strains), and GII.P10 (2 strains). The phylogenetic analyses are shown in Figure 3. Combined genotyping of the VP1 and RdRp regions identified 66 strains that were successfully detected in both regions, of which GI.1[P1] was the most common (37.88%, 25/66).

In 2022, five genotypes and five P-genotypes were identified, with GI.1 and GI.2 being the most frequently detected in VP1 region and GI.P1 being the most common in RdRp region. In 2023, eight genotypes and eight P-genotypes were detected. The most frequently identified genotypes were GI.1 and P-genotypes were GI.P1. The details are presented in Appendix A Table A1.

### 3.4. Analysis of Amino Acid Variation Characteristics

Given the scarcity of complete GI.3 SaV genomes in GenBank (only eleven available), metagenomic sequencing was performed on two GI.3 SaV strains obtained in this study, with only one successfully sequenced. Amino acid variation was analyzed between the strain and all 11 reference sequences of GI.3 type retrieved from GenBank. Among the proteins encoded by ORF1, NS1 was the region most prone to amino acid variation (5.88%, 4/68), followed by NS6-NS7 (3.74%, 25/668), NS5 (2.63%, 3/114), NS2 (2.34%, 6/256), NS3 (1.17%, 4/341), VP1 (0.88%, 5/565), and NS4 (0.73%, 2/274). In the VP2 region encoded by ORF2, 5.45% of amino acid variation was observed (9/165). The results are shown in Appendix A, Table A2 and Table 5.

## 4. Discussion

Though studies on gastroenteritis viruses have mostly focused on norovirus and rotavirus, SaV is a key pathogen responsible for sporadic infection and outbreaks of acute gastroenteritis in humans [3]. In a study conducted in Japan by Hoque et al., the SaV positivity rate was 8.30% [8]. In a subsequent study by Li et al., the SaV positivity rate was 2.77% [13]. A separate study in Spain reported a SaV positivity rate of 8% [14]. Our study recorded a SaV positivity rate of 1.12% in Shandong during 2022–2023, which is relatively low compared to previous studies. Countries such as Japan, Spain, and Burkina Faso have incorporated the rotavirus vaccine into their national immunization programs, which likely contributes to the increasing prominence of their higher SaV positivity rates. In addition, this study covered the whole age group, while other studies mostly covered children, and our study also found that the positive rate in children aged 3–5 was significantly higher than that in other age groups. It is worth mentioning here that SaV can infect both preschool-aged children and the elderly. Young children, particularly those under the age of five, are highly susceptible due to their immature immune systems and frequent exposure to communal environments such as daycare centers. The SaV positivity rate in 2023 was significantly higher than that in 2022. In late 2022, changes in COVID-19 control strategies in Shandong Province may have contributed to an altered SaV epidemiological landscape in 2023, resulting in increased detection rates.

SaV was detected throughout the year, but in this study, no cases were reported in April 2022, possibly due to the severe precautionary measures because of the COVID-19 outbreak in Shandong Province from March to May 2022. In both 2022 and 2023, the highest SaV positivity rates were observed in November, consistent with the findings of Ji et al. [15]. This was earlier than the peaks observed by Hoque et al. and Li et al. [8,13]. But all studies showed the peaks occurred in cold months, aligning with the study by Dey et al. [16]. It is recommended that medical institutions adopt a more targeted testing approach.

This study examined the prevalence of SaV in 16 cities across Shandong Province during 2022–2023. The highest SaV positivity rate in 2022 was observed in Dongying City, which was explained by the effective control of COVID-19 that allowed for normal population movement. In addition, Zaozhuang City recorded the highest SaV positivity rate in 2023. This study also highlighted fluctuations in SaV positivity rates across different cities over time, emphasizing the importance of ongoing viral diarrhea surveillance to monitor long-term trends.

SaV infection is considered mild, but our study showed no significant difference in SaV positivity rates between outpatient and inpatient cases. The number of SaV-positive cases in this study was relatively low; therefore, expanded surveillance is required to better assess the severity of clinical symptoms. A comparison between SaV-positive and SaV-negative patients in this study revealed that vomiting was a distinctive symptom associated with SaV infection. This is consistent with the results of Tang et al. [17]. It is suggested that more attention be paid to vomiting symptoms in SaV surveillance in the future. SaV is characterized by diverse transmission modes, high viral shedding, and low infectious doses [18]. Proper handling of vomitus from SaV-infected patients is essential to prevent these materials from becoming risk factors for outbreaks, which could lead to significant public health concerns.

The adoption of a dual-typing method provides a more comprehensive understanding of SaV sequences [10]. In this study, a dual-typing method was used based on the VP1 and RdRp nucleotide sequences. The predominant genotype in Shandong was identified as GI.1[P1], which aligns with findings from several regions where GI.1 is also the dominant genotype [19,20,21,22]. Furthermore, this study identified three additional genotypes, i.e., GI.3, GII.1, and GIV.1 in 2023 compared to 2022, reflecting an increase in genotype diversity. The complete genome sequence of a GI.3 strain revealed that NS1 was the region most prone to amino acid variation, followed by VP2, which is consistent with the result for GI.1 SaV [23]. Considering that most of the current studies are on VP1, future studies should pay more attention to other regions, and SaV surveillance based on whole-genome sequencing is essential.

In summary, we studied 14,011 cases of gastroenteritis across all age groups during and after the COVID-19 epidemic. An upward trend and an increase in the genotype diversity of SaV were observed after the COVID-19 epidemic in our study. In addition, with the wider use of the rotavirus vaccine in Shandong, it is essential to conduct continuous surveillance on SaV.

## Figures and Tables

**Table 1:** Monthly distribution of SaV infection cases.

	2022			2023		
Month	Cases (N)	Positive Cases (N)	SaV Positivity Rate (%)	Cases (N)	Positive Cases (N)	SaV Positivity Rate (%)
1	417	2	0.48	471	2	0.42
2	538	5	0.93	632	4	0.63
3	489	1	0.20	638	5	0.78
4	465	0	0	649	10	1.54
5	581	2	0.34	714	10	1.40
6	701	7	1.00	763	11	1.44
7	655	9	1.37	741	12	1.62
8	582	4	0.69	734	12	1.63
9	530	5	0.94	659	8	1.21
10	499	3	0.60	674	11	1.63
11	466	7	1.50	570	15	2.63
12	367	2	0.54	476	10	2.10
Total	6290	47	0.75	7721	110	1.42

**Table 2:** Detection of SaV in different age groups.

Age (Years)	Cases (N)	Positive Cases (N)	SaV Positivity Rate (%)	*χ^2^*	*P*-Value
0–2	3809	56	1.47	47.289	<0.05
3–5	1394	33	2.37		
6–11	923	13	1.41		
12–17	689	13	1.89		
18–59	4461	29	0.65		
≥60	2735	13	0.48		
Total	14,011	157	1.12		

**Table 3:** Comparison of SaV detection rates between outpatients and inpatients.

Type of Medical Visit	Cases (N)	Positive Cases (N)	SaV Positivity Rate (%)	*χ^2^*	*P*-Value
Outpatients	11,458	125	1.09	0.580	0.446
Inpatients	2358	30	1.27		
Total	13,816	155	1.12		

**Table 4:** Comparison of clinical symptoms between SaV-positive and SaV-negative patients.

Clinical Symptoms	SaV-Positive	SaV-Negative	*χ^2^*	*P*-Value
Fever				
Yes	41	2892	2.575	0.109
No	116	10,962		
Nausea				
Yes	32	2890	0.022	0.883
No	125	10,964		
Vomiting				
Yes	53	3227	9.482	0.002
No	104	10,627		
Diarrhea				
Yes	147	13,240	1.369	0.242
No	10	614		
Abdominal pain				
Yes	53	5377	1.671	0.196
No	104	8477		

**Table 5:** Amino acid variation of ORF2 in genotype GI.3.

	VP2								
	31	46	137	138	149	150	151	159	165
SD20230757	A	T	S	A	N	T	G	S	V
LC504389		I	P					T	
LC504390	T		P					T	
LC504391			P						
LC504392			P	T				T	
LC504393		I	P					T	
LC504394		I	P					T	
LC504395		I	P				C	T	
AB623037			P					T	
AB522396		I	P		S				A
MG012401			P						A
PQ510869			P			I			

## Data Availability

The sequences obtained in this study are available at the NCBI GenBank repository with accession number PV014972.

## Appendix A

### Appendix A.1

**Table A1:** Genotype distribution by year.

Year	Genotype	Case (N)	Composition Ratio (%)	P-Genotype	Case (N)	Composition Ratio (%)	Dual Genotype	Case (N)	Composition Ratio (%)
2022	GI.1	8	30.77	GI.P1	7	35.00	GI.1[P1]	7	38.89
	GI.2	8	30.77	GI.P2	5	25.00	GI.2[P2]	4	22.22
	GI.6	1	3.85	GI.P6	1	5.00	GI.6[P6]	1	5.56
	GII.3	6	23.08	GII.P3	6	30.00	GII.3[P3]	5	27.78
	GII.5	3	11.54	GII.P5	1	5.00	GII.5[P5]	1	5.56
Total		26	100.00		20	100.00		18	100.00
2023	GI.1	20	31.25	GI.P1	18	37.50	GI.1[P1]	18	37.50
	GI.2	7	10.94	GI.P2	5	10.42	GI.2[P2]	5	10.42
	GI.3	2	3.13	GI.P3	2	4.17	GI.3[P3]	2	4.17
	GI.6	7	10.94	GI.P6	4	8.33	GI.6[P6]	4	8.33
	GII.1	10	15.63	GII.P1	10	20.83	GII.1[P1]	10	20.83
	GII.3	4	6.25	GII.P3	4	8.33	GII.3[P3]	4	8.33
	GII.5	11	17.19	GII.P5	3	6.25	GII.5[P5]	3	6.25
	GIV.1	3	4.69	GII.P10	2	4.17	GIV.1[GII.P10]	2	4.17
Total		64	100.00		48	100.00		48	100.00

### Appendix A.2

**Table A2:** Amino acid variation of ORF1 in genotype GI.3.

	NS1				NS2						NS3				NS4		NS5			NS6-NS7																									VP1				
	42	49	55	64	96	105	199	223	314	319	375	409	560	642	700	932	983	1044	1052	1096	1111	1123	1129	1204	1205	1207	1221	1243	1305	1340	1342	1353	1362	1367	1400	1440	1490	1514	1533	1573	1588	1607	1635	1646	1735	1944	1994	1997	2232
SD20230757	F	S	A	R	A	T	T	E	I	S	I	E	S	N	S	S	D	A	E	P	T	D	F	T	V	S	I	P	R	E	I	D	V	A	V	T	I	I	E	G	D	P	A	S	T	K	A	V	V
LC504389		G	V							T						T					A	G									A							V				A	T					I	
LC504390																T					A	G			I						A							V				A	T						
LC504391																					A	E																				A							
LC504392					T											T	E				A	E		A							A							V				A	T	G					
LC504393	S		V							T						T					A	G									A							V				A	T					I	
LC504394		G	V							T						T					A	G									A							V				A	T					I	
LC504395		G	V							T						T		V			A	G									A							V				A	T					I	
AB623037																T					A	G									A							V				A	T						
AB522396	L		V	K	T	A		D			V	D			N	T					A	E	Y						K	D	T				I			V	D	S		A	T		I	R			
MG012401	S				P	A								T		T			A	S	A	E				N	V		K		T	E	I	V			V	V		S	E	A	T				S		I
PQ510869			I				T		V				S															S								A													
